# Evaluation of pulmonary function among detergent powder factory workers- a cross sectional study in Semnan, Iran

**DOI:** 10.1186/s12995-022-00347-1

**Published:** 2022-02-09

**Authors:** Farhad Malek, Elham Ranjbari, Majid Mirmohammadkhani, Daryoush Pahlevan

**Affiliations:** 1 Department of Internal Medicine, Kowsar Hospital, Semnan University of Medical Sciences, Semnan, Iran; 2 Student Research Committee, Semnan University of Medical Sciences, Semnan, Iran; 3 Determinants of Health Research Center, Semnan University of Medical Sciences, Semnan, Iran

**Keywords:** Occupational disease, Spirometry, Detergents, Lung disease

## Abstract

Occupational respiratory diseases are the most prevalent occurring work-related diseases that contribute to global health concerns. The present study aimed to assess pulmonary function among detergent powder factory workers.

In a cross-sectional study, 305 employees working at a detergent powder company in Semnan, Iran were enrolled. Demographic characteristics, health- and job-related information were recorded using a checklist. Subsequently, spirometer was used at baseline, before and after shift-working for recording respiratory ailments and pulmonary function tests (PFT).

According to the results, the mean percentage of all spirometric indices significantly reduced after shift-work including forced vital capacity (FVC) (*P* < 0.01), forced expiratory volume in one second (FEV1) (*P* < 0.01), FEV1/FVC ratio (*P* = 0.038), peak expiratory flow (PEF) (*P* = 0.13) and forced expiratory flow at 25 and 75% of the pulmonary volume (FEF (25–75)) (*P* < 0.01). Although the mean percentage of FEV1 significantly improved upon wearing the protective mask (*P* = 0.014). Moreover, FVC and FEV1 indices were significantly less in smoking workers than in non-smoking participants (*P* = 0.005 and *P* = 0.003, respectively).

This study revealed that using effective preventive measures should be tightly performed to promote health conditions. However, despite the occupational health programs for preventing and reducing work-related respiratory diseases, these can be considered as a serious threat for detergent powder factory workers that need to apply more control strategies and health assessment.

## Introduction

With worldwide increases in production and consumption through the increasing population, occupational exposure is a substantial global health concern. Indeed, occupational disease is a major cause of disability and absence from work in the working population [1]. According to the World Health Organization, approximately 68–157 million new cases of occupational disease are attributed to hazardous exposures or workloads with more than 200,000 death, annually [2]. However, workers in low- and middle-income countries are more exposed to the work-related diseases. According to the Global Burden of Disease study conducted in 2010 (GBD 2010), occupational exposures are the ninth major cause of disability-adjusted life years (DALYs) in Iran [3]. The International Labor Organization has provided a list of occupational diseases, including diseases caused by biological, physical and chemical agents, respiratory disorders, skin diseases, musculoskeletal diseases, also mental and behavioral disorders, and occupational cancers [4]. Particularly, one-third occupational disease consisted of interstitial lung disease and respiratory cancers [5]. Although the impressive part of occupational lung diseases is not systematically recorded and the current statistics underestimates the true burden, up to 25% of all lung cancer deaths are attributed to causes of occupational exposure. In Iran, occupational exposure accounted for 13% of chronic obstructive pulmonary disease (COPD), 11% of asthma and 9% of lung cancers [3]. A variety of acute and chronic pulmonary diseases are caused by inhaling hazardous chemical agents at the workplace. The exposure to dusts, vapors, immunological agents and microscopic airborne in the workplace are associated to occupational lung diseases [6].

In recent decades, using cleansing products has widely increased, partly due to environmental reasons. Manufacturing of these products has comprised the chemicals that continuously replaced by new ingredients to improve cleaning efficacy. Several methods are used to produce powder detergents, including spray drying, agglomeration, dry mixing, or combinations of these methods. The aim of these methods is the production of small droplets along with the generation of detergent dust and air pollution. Therefore, occupational respiratory diseases caused by proteolytic enzymes, surfactant, alkaline ingredients and bleaches are reported in the detergent industry. Moreover, the detergent powder manufacturing industry is associated with a risk of silica emission and its consequences. Silicosis as the most common occupational lung disease, is caused by continuous exposure to dust containing free silica [7].

The immediate hypersensitivity, asthma, and rhinitis are the most common occupational respiratory diseases of detergent workers [8, 9]. It is demonstrated that the risk of developing occupational asthma is related to the intensity of exposure to the causative agent and early detection can lead to appropriate prevention and treatment. To identify work-related respiratory disorders, after explaining biography and physical examination, various methods including chest x-ray, immunological studies, biomarker measurements, spirometry, etc. can be used. Spirometry is most applicable to discover the pulmonary impairment and also is one of the most important screening tools in pulmonary diseases [10]. Spirometry is the cornerstone of prevention and treatment of workplace-related lung disease, that is used to identify workers who should have further evaluation for possible disease [11]. Based on the important impacts of spirometry collected data for the occupational health specialist and health care physicians, the aim of this study was the evaluate spirometry parameters of detergent powder factory workers at Kondor industry, Semnan, Iran.

## Methods

### Study design and participants

In this cross-sectional study, the statistical population consisted of 305 workers at Kondor Powder Company in Semnan, Iran, who met the eligibility criteria (inclusion criteria; have a complete personal and health records and exclusion criteria; lack of cooperation and also have any underlying disease that can affect spirometry results, including a history of asthma, bronchiolitis, cystic fibrosis, hypersensitivity pneumonitis, chronic bronchitis upon treatment with bronchodilators) were enrolled. The detergent powders are composed of sodium silicate and enzymes, including proteases, lipases, amylases, and celluloses with pH 7.8. This study was approved by the local ethics committee (92.338134; 1392.06.05) and written informed consent was taken from participants.

### Anthropometric measurements

Along with personal information (age and gender), anthropometric (height (centimeters), weight (Kgs) and Body Mass Index (BMI) (according to the Quetelet formula) [12], respiratory health history, description of different types of occupational exposures, work experience, job function and underlying disease were also recorded.

### Pulmonary function tests

The current study was performed according to the American Thoracic Society/European Respiratory Society (ATS/ERS) guideline recommendations using spirometry. At first, participants were introduced regarding the spirometer principal, operation and procedure. The participant’s data, including name, age, height, weight and date of testing were recorded in the SpiroLab II spirometer (MIR, Rome, Italy). Subsequently, spirometry was carried out in standing position by wearing nose clip while the subjects taking full inspiration and rapid forceful expiration in the mouthpiece of instrument, before and after shift-work, and data (including forced vital capacity (FVC), forced expiratory volume in one second (FEV1), FEV1/FVC ratio, peak expiratory flow (PEF) and forced expiratory flow at 25 and 75% of the pulmonary volume (FEF _(25–75)_)) were recorded. For every participant two spirometry reading were conducted; before and after shift-work (with 8 h’ interval). Moreover, any possible contraindications to spirometry was considered before and during the spirometry.

### Statistical analysis

The data analysis was performed using Statistical Packages for Social Sciences (SPSS) software version 18.0 (Chicago, USA). Also, paired t-test and ANOVA were employed for comparing the spirometry parameters, before and after shift-work and comparison between subgroups. *P*- value < 0.05 is considered as significant.

## Results

### Demographic characteristics and impact on spirometry parameters

Of 305 participants were included in this cross-sectional study, 290 (95.1%) were male and 15 (4.9%) were female. The participants mean age was 35.03 ± 6.67 (mean ± standard deviation) with the minimum age of 25 years old and maximum age of 65 years old. Moreover, more than 85% of participants had no history of smoking and allergic diseases. The main demographic and anthropometric characteristics of participants and differences between before and after of pulmonary function parameters are summarized in Table 1. Subjects did not differ statistically in terms of the males and females, age (< 35 years old and ≥ 35 years old), BMI (≤ 25 and > 25) or the history of allergic diseases. However, there were the significant differences of FVC and FEV1 parameters between smoking and non-smoking participants (*P* = 0.005 and *P* = 0.003, respectively).

**Table 1:** Demographic, anthropometric and pulmonary function parameters of participants

Variables	Number (%)	Pulmonary function parameters									
		ΔFVC (%)	*P* value	ΔFEV1 (%)	*P* value	ΔFEV_1_/FVC(%)	*P* value	ΔPEF (%)	*P* value	ΔFEF _(25–75)_(%)	*P* value*
**Age**											
< 35 years old	162 (53.1%)	7.76 ± 1.95	0.52	7.60 ± 2.05	0.86	5.16 ± 0.43	0.49	10.72 ± 0.35	0.31	17.6 ± 6.70	0.14
≥ 35 years old	143 (46.9%)	7.75 ± 1.37		8.40 ± 2.20		5.25 ± 0.83		11.7 ± 1.65		21.4 ± 9.96	
**Sex**											
Male	290 (95.1%)	7.7 ± 1.63	0.63	8.01 ± 2.06	0.56	5.3 ± 0.60	0.81	10.97 ± 0.98	0.87	19.76 ± 8.42	0.46
Female	15 (4.9%)	9 ± 2.6		7.04 ± 3.26		2.8 ± 0.93		15.25 ± 0.53		13.75 ± 8.66	
**BMI**											
≤ 25	158 (51.8%)	8.3 ± 0.98	0.10	8.06 ± 1.72	0.37	5.12 ± 0.94	0.26	12.66 ± 0.16	0.06	18.22 ± 6.95	0.23
> 25	147 (48.2%)	7.05 ± 2.43		7.23 ± 2.55		5.27 ± 0.27		9.25 ± 2.18		20.08 ± 9.60	
**Smoking**											
Yes	43 (14.1%)	7.9 ± 1.4	0.005	9.95 ± 1.20	0.003	6.20 ± 0.35	0.70	14.4 ± 0.82	0.30	33.20 ± 10.60	0.40
No	262 (85.9%)	7.62 ± 2.20		7.5 ± 2.70		5.03 ± 0.66		10.60 ± 1.25		16.30 ± 7.85	
**History of allergic disease**											
Yes	45 (14.8%)	8.40 ± 1.90	0.86	8.50 ± 1.90	0.84	5.60 ± 0.73	0.058	12.50 ± 0.50	0.75	16.30 ± 6.20	0.45
No	260 (85.2%)	7.70 ± 1.65		7.90 ± 2.15		5.10 ± 0.85		11 ± 10.4		20.02 ± 8.06	

*BMI* body mass index, *FVC* forced vital capacity, *FEV1* forced expiratory volume, *PEF* peak expiratory flow, *FEF*_*(25–75)*_ forced expiratory flow at 25 and 75% of the pulmonary volume; Values are the mean ± standard deviation. Δ; differences between before and after of shift-work

*Paired t-test and ANOVA

### Respiratory health condition and occupational variables

Subsequently, spirometry parameters were assessed in variety of occupational factors before and after of 8- h shift-work. As noted in Table 2, FEV1 showed a significant difference between workers who wore mask in the workplace and who had no mask (*P* = 0.014). However, none of the job responsibility, work experience and neither work-related risk factors had significant impacts on spirometry parameters.

**Table 2:** Occupational factors and pulmonary function parameters of participants

Variables	Number (%)	Pulmonary function parameters									
		ΔFVC (%)	*P* value	ΔFEV1 (%)	*P* value	ΔFEV_1_/FVC(%)	*P* value	ΔPEF (%)	*P* value	ΔFEF _(25–75)_(%)	*P* value*
**Job title**											
Administrative staff	17 (5.6%)	6.61 ± 2.53	0.87	7.7 ± 3.94	0.57	4.55 ± 0.59	0.70	12.61 ± 0.59	0.82	19.47 ± 4.23	0.60
Driver	15 (4.9%)	5.48 ± 1.20		6. 04 ± 1.20		5.72 ± 047		6.74 ± 2.67		16.25 ± 10.87	
Worker	273 (89.5%)	7.93 ± 1.65		8. 07 ± 2.06		5.22 ± 0.67		11.31 ± 0.90		19.71 ± 8.34	
**Work experience**											
≤ 7 years	157 (51.5%)	8.14 ± 1.92	0.57	8.58 ± 2.24	0.79	5.32 ± 0.71	0.75	12.45 ± 0.24	0.24	16.21 ± 8.20	0.93
> 7 years	148 (48.5%)	7.31 ± 1.42		7.27 ± 2.00		5.09 ± 0.52		9.67 ± 1.73		22.55 ± 8.32	
**Using mask**											
Never	63 (20.7%)	8.4 ± 0.65		8.07 ± 1.22		3.45 ± 0.75		12.90 ± 1.50		26.65 ± 11.15	
Sometimes	35 (11.5%)	4.30 ± 2.82	0.38	5.80 ± 4.85	0.014	5.50 ± 3.20	0.70	6.08 ± 6.70	0.005	17.40 ± 9.70	0.002
Usually	207 (67.9%)	8.00 ± 1.80		8.20 ± 1.80		5.50 ± 0.14		11 ± 0.85		17.70 ± 5.80	
**Exposed to environmental risk factors**											
Exposed	240 (78.7%)	7.70 ± 1.50	0.48	1.90 ± 17.90	0.36	5.40 ± 0.67	0.72	11.20 ± 0.90	0.90	19.90 ± 8.45	0.71
No exposure	65 (21.30%)	8.09 ± 2.30		2.90 ± 8.09		4.50 ± 0.41		11.1 ± 1.10		18.25 ± 7.45	

*FVC* forced vital capacity, *FEV1* forced expiratory volume, *PEF* peak expiratory flow, *FEF*_*(25–75)*_ forced expiratory flow at 25 and 75% of the pulmonary volume; Values are the mean ± standard deviation. Δ; differences between before and after of shift-work

*Paired t-test and ANOVA

### Pulmonary functions parameters before and after shift-work

Table 3 displays different spirometry observed values at baseline, before and after of shift-work. According to the third National Health and Nutrition Examination Survey (NHANES III) equations [13] there were the significant differences between before and after shift-work in all parameters, including FVC (*P* < 0.01), FEV1 (*P* < 0.01), FEV1/FVC (*P* = 0.038), PEF (*P* = 0.13) and FEF _(25–75)_ (*P* < 0.01).

**Table 3:** The pulmonary function parameters at baseline, before and after of shift-work (*n* = 305)

Pulmonary function Parameters (%)	Baseline (Predicted)	Before shift	After shift	Total Changes (%) (Mean ± SD)	***P*** value*
**FVC**	96.80	98.00	96.40	1.7 ± 7.75	< 0.001
**FEV1**	95.85	94.95	92.80	2.2 ± 7.96	< 0.001
**FEV1/FVC**	82.25	90.95	90.30	0.62 ± 5.20	0.038
**PEF**	99.15	100.15	99.20	0.97 ± 11.20	0.13
**FEF**_**(25–75%)**_	86.60	91.40	83.20	8.24 ± 19.50	< 0.001

*FVC* forced vital capacity, *FEV1* forced expiratory volume, *PEF* peak expiratory flow, *FEF (25–75)* forced expiratory flow at 25 and 75% of the pulmonary volume. Values are the mean ± standard deviation

*Paired t-test and ANOVA

## Discussion

Occupational-related respiratory diseases account for 10–20% of all chronic respiratory disorders. Detergent powder industries are often associated with hazardous working conditions for respiratory disorders. In the present study, for the first time, the spirometric indices were assessed before starting shift-work and at the end of 8 h shift-work in Kondor detergent powder company in Semnan, Iran. The results suggested that there are no significant differences of spirometry parameters in workers with various demographic characteristics. Although the extent of pulmonary function loss tended to be higher among workers with BMI ≥25, it was not statistically significant. Among of these demographic parameters, smoking significantly altered FVC and FEV1 parameters. Smoking is regarded an undeniable risk factor that triggers lungs diseases. According to the body of evidence, FVC, FEV1, and FEV1/FVC% were found to be lower in smokers than non-smokers [14]. In line with our results, Maritta et al, demonstrated that smoking has a synergistic effect to dust on lung function of tile factory workers [15]. In this regard, Attarchi et al. reported a significant synergistic effect of cigarette smoking and occupational exposures on lung function of rubbery workers [16]. These findings revealed that cigarette smoking has been implicated as a risk factor for respiratory disease among industry workers. Our results showed that not only workers who were in direct exposure to chemical agents and detergents, but also other employees including administrative staffs exhibited the signs of loss of pulmonary function.

Moreover, Ibraheem TM et al. reported that FEV1, FVC and PEF are significantly reduced among exposed detergent workers [17]. However, respiratory protective equipment such as mask can reduce the incidence of occupational diseases. In this line, our investigation of the workplace variables also showed that using protective mask improves FEV1. Leslie et al. concluded that 80% reduction in occupational asthma due to hexahydrophthalic anhydride (HHPA), mainly attributed to the respiratory protection [18]. This finding has clinical significance from a societal perspective.

It is demonstrated that enzymes used in the detergent factories may have the potential to sensitize respiratory system. Cullinan et al. reported that the highest duration of exposure to proteases -one of the four types of enzymes used in detergents- led to lower respiratory diseases, while the low exposure increased the risk of upper respiratory diseases [5]. Many studies have proved a respiratory disorder during long exposure to chemical hazards such as dust, exhausts, fuels and detergents. Our results showed a statistically significant lowered mean of FVC, FEV1, FEV1/FVC, PEF and FEF _(25–75)_. According to the Chakraborty et al. report, laundry workers exhibited more respiratory symptoms and abnormal spirometry parameters due to prolonged exposure to noxious related-particles [19]. In line with our results, a cross-sectional study was carried out on detergent product staff showed both restrictive lesions (52%) and obstructive lesions (2.4%) with more than 65% radioallergosorbent testing (RAST) grade III. Moreover, RAST grade III workers also revealed a reduced pulmonary function including FVC and FEV1 [17].

The limitation of the current study is using only cross-shift measurement without considering daily respiratory symptoms and the lack of longitudinal follow-up to determine long-term complications upon work- place exposure. However, lack of conduction of bronchodilator reversibility test may be another limitation of the study.

## Conclusion

In conclusion, in this study showed that detergent powder factory workers significantly exhibited loss of pulmonary function after shift-work, a health threatening situation that needs primary prevention, including intermittent-workplace education courses, exposure reduction and control, and also elimination of hazardous agents. Together, despite the cooperative efforts to prevent and control occupational pulmonary disorders, the more effective health guidelines and methodology need to be developed. In this regards, requirement of regular health condition assessments such as spirometry are essential in at-risk worker population.

## Data Availability

All raw and analyzed data are accessible.

## Abbreviations

FVC: Forced vital capacity
FEV1: Forced expiratory volume in one second
PEF: Peak expiratory flow
FEF _(25–75)_: Forced expiratory flow at 25 and 75% of the pulmonary volume
